# Obturator nerve endometriosis: A systematic review of the literature

**DOI:** 10.52054/FVVO.14.3.032

**Published:** 2022-09-30

**Authors:** A Kale, Y Aboalhasan, E.C. Gündoğdu, T Usta, E Oral

**Affiliations:** Department of Obstetrics and Gynecology, University of Health Science Kartal Dr Lutfi Kirdar City Hospital, Istanbul, Turkey; Department of Obstetrics and Gynecology, Siirt Training and Research Hospital, Siirt, Turkey; Department of Obstetrics and Gynecology, Acibadem University, Altunizade Hospital, Istanbul, Turkey; Department of Obstetrics and Gynecology, Bezmialem Vakif University, Istanbul, Turkey

**Keywords:** endometriosis, deep infiltrating endometriosis, obturator nerve, laparoscopy

## Abstract

**Background:**

Symptomatic obturator nerve endometriosis is a rare condition. In this paper, we aim to review and discuss the characteristics of obturator nerve endometriosis in light of current literature.

**Methods:**

An electronic search was conducted using the PubMed/Medline database.

**Results:**

Symptomatic obturator nerve endometriosis is rare; only 8 cases have been reported in the literature. Symptoms including difficulty walking, weak thigh adduction and pain in the inner thigh, which are all related to obturator nerve function, could be seen in the case of the entrapment of the nerve by endometrial nodules. A history of recurrent symptoms during menstrual cycles and physical examination, combined with appropriate radiologic imaging, led to a suspicion of obturator nerve involvement.

**Conclusion:**

Early diagnosis and surgical treatment of obturator nerve endometriosis is essential to minimise the nerve damage caused by recurrent cycles of bleeding and fibrosis, which are characteristics of endometriosis. The laparoscopic minimally invasive technique is feasible for the surgery of obturator nerve endometriosis. It offers the advantage of precise discrimination of vital structures and excellent access to deep anatomic sites.

**What's new?:**

Obturator nerve endometriosis may be a severe cause of chronic pelvic pain in women of reproductive age. Treatment may be achieved surgically and in experienced hands, laparoscopic surgery would be the preferred choice.

## Introduction

Endometriosis is a chronic, inflammatory disease classically defined as the presence of endometrial glands and stroma outside the uterine cavity (Api, 2015; Berker and Seval, 2015). Endometriosis can present with various signs and symptoms, including dysmenorrhea, dyspareunia, dyschezia, dysuria, and chronic pelvic pain (Della Corte et al., 2020).

Deep infiltrating endometriosis (DIE) is a particular form of endometriosis that may cause severe pelvic pain in women. The aggressive and invasive nature of DIE may lead to the involvement of peripheral nerves. The most common involved site is the sacral plexus (57%), followed by the sciatic nerve (39%) (De Sousa et al., 2015). There are few reported cases of involvement of the other pelvic nerves, such as the obturator nerve (<1%) (Kondo et al., 2013; Koninckx and Martin, 1994; Koninckx et al., 1991; Bradshaw and McCrory, 1997; Redwine and Sharpe, 1990; Langebrekke and Qvigstad, 2009; Osorio et al., 2018; Waer et al., 2012). Pain is the most prominent and frequent symptom (97%) (Stratton and Berkley, 2011). Numbness of the skin (31%) and weakness of the muscle group (20%) supplied by the affected nerve may also exist (De Sousa et al., 2015).

Symptomatic obturator nerve endometriosis is rare, and only 8 cases have been reported in the literature. This review article aims to discuss the characteristics of obturator nerve endometriosis in light of current literature.

## Methods

In January 2022, an electronic literature research was conducted using a PubMed/Medline search on the keywords ‘obturator nerve endometriosis’, ‘obturator nerve entrapment,’ ‘endometriosis along the obturator nerve’ in combination with the Boolean operators AND and OR. In addition, the references of the returned articles were also searched for relevant citations. This review included all eight founded case reports (Between 1990-2019). A comparison of all the reported cases is shown in Table I.

**Table I:** Main features of reported cases of obturator nerve endometriosis in literature.

Author	Year	Age	Symptom	Imaging	Findings	Medical Treatment	Surgical Treatment	Follow Up	Recurrence
Redwine et al.	1990	33	Pain in the inner right thigh and proximal right leg weakness	Unreported	Unreported	None	Laparotomy	Unreported	None
Ekpo et al.	2007	33	Left lower extremity pain and difficulty ambulating, particularly weakness with adduction of the thigh	MRI	Two 7-mm endometrial deposits at the right posterior aspect of the uterus. A 3.7x3.8-cm cystic lesion in the right adnexa consistent with an endometrioma. Two large cystic structures found in the left adnexa, a 5.2x2.7-cm endometrioma extending into the retroperitoneum and a 6.3x3.4-cm structure believed to be a hydrosalpinx	None	Laparoscopy	2 weeks	None
Lange-brekke et al.	2009	26	Increasing pain and impaired adduction of the right leg	MRI	An endometriosis-like structure detected close to the right obturator nerve.	None	Laparoscopy	3 months	None
Fambrini et al.	2010	45	Deep pain in the left thigh while walking	MRI	An infiltrated area measuring 8.7x6.6 cm between the adductor magnus and gracilis muscles.	None	Laparotomy	6 months	None
Waer et al.	2012	30	Undulating pain and swelling located in the right thigh fluctuated with her menstrual cycle	MRI	Polynodular 5x3x2 cm structure originating from the intrapelvic region, posterolateral to the right ovary and adjacent to the incisura ischiadica major, where it touched the sciatic nerve. It extended along the obturator nerve through the obturator foramen into the right adductor thigh compartment	Hormonal Therapy	Laparotomy	Unreported	None
Osorio et al.	2018	32	Cyclic leg pain in the inner right thigh, radiating to the knee	MRI	A likely endometriotic nodular lesion of about 2.3 cm, inside the right obturator internus muscl.	None	Laparoscopy	6 months	None
Kalkan et al.	2018	25	Chronic, non-cyclic, severe inguinal pain, more prominent in the inner right thigh	MRI	A nodular, solid, retractile, fibrotic-like hyperintense 2 cm mass lateral and inferior to the right ovary. The mass was extending inferiorly to the pelvic floor as a more hyperintense structure with intermingled hypointense foci consistent with endometriosis.	None	Laparoscopy	18 months	None
Peters et al.	2019	32	Pelvic pain	Unreported	Unreported	None	Laparoscopy	Unreported	None

## Symptoms and diagnosis

DIE nodules may involve the pelvic nerves, mainly when there is infiltration of the parametrium. Parametrial nodules may compress, surround or infiltrate nerves of large diameters, such as sacral roots, sciatic or pudendal nerves; or thin fibres, such as splanchnic or hypogastric nerves in the inferior or superior hypogastric plexus. Depending on the role of nerve fibres, somatic and vegetative symptoms may occur. These may include pain in buttocks, legs or perineum, skin hypo or hyperesthesia, bladder, rectal dysfunction or vaginal dryness (Lemos et al., 2015).

Entrapment of the obturator nerve due to DIE is extremely rare (<1% among the involved peripheral nerves by DIE implants), (Kondo et al., 2013; Koninckx and Martin, 1994; Koninckx et al., 1991; Bradshaw and McCrory, 1997; Redwine and Sharpe, 1990; Langebrekke and Qvigstad, 2009; Osorio et al., 2018; Waer et al., 2012). The obturator nerve is responsible for motor and sensitive innervation of the joints and internal muscles of the thigh and knee, and the innervation of the inner thigh skin. In addition to the pelvic pain seen in DIE, pain along the sensitive area of the obturator nerve, thigh adduction weakness, difficulty in ambulating, or paraesthesia can be present with the involvement of the obturator nerve (Ekpo et al., 2007).

The diagnosis of extra pelvic endometriosis may be challenging. The sonographic appearance of perineural endometriosis is usually nonspecific. Magnetic resonance imaging (MRI) is the first and preferred modality for evaluating endometriosis involving the pelvic nerve roots. MRI has high sensitivity (90%) and specificity (98%) with direct visualisation of nerve roots. On MRI, endometriosis appears as solid nodules or as a focal lesion with cystic components and variable signal intensities due to the presence of blood products. With MRI, it is also possible to evaluate the perineural spread of endometriosis seen as nerve thickening, widening neural foramen and loss of the fat surrounding the nerve (Lomoro et al., 2019).

## Surgical treatment of obturator nerve endometriosis

The laparoscopic minimally invasive technique is feasible for the surgery of DIE. It offers the advantage of precise discrimination of vital structures and good access to deep anatomical sites (Kondo et al., 2013). Surgical experience and knowledge of retroperitoneal anatomy are critical to managing DIE as it is a disease that is rarely confined to the surface. Laparoscopic excision and clearing the endometriotic and dense fibrotic tissue are as follows: 1) Dissection of peritoneum and access to retroperitoneum starting from the border of the pelvic brim, 2) Ureterolysis, 3) Exposure of the internal iliac vessels, 4) Exposure of the entrapped obturator nerve, 5) Careful stripping of the fibrotic tissue surrounding the obturator nerve using both sharp and blunt dissection without causing injury to the nerve. (Kalkan et al., 2018).

## Discussion

DIE is a particular form of endometriosis defined as lesions that penetrate >5 mm under the peritoneal surface (Koninckx and Martin, 1994). These lesions are considered very active and are strongly associated with pelvic pain symptoms in women of reproductive age (Koninckx et al., 1991) and may involve almost every organ. Aggressive infiltration of the retroperitoneal space with neural involvement such as the obturator nerve is less common. This nerve and its branches supply the muscle and skin of the medial thigh. In addition to the commonest symptom of pelvic pain seen with endometriosis, weakness in thigh adduction or difficulty ambulating may also present with entrapment of the obturator nerve (Bradshaw and McCrory, 1997).

According to our search, only 8 cases of symptomatic obturator nerve involvement by DIE have been reported between 1990-2019. Almost all the reported patients had symptoms relating to obturator nerve endometriosis. These include worsening walking ability, difficulty in thigh adduction, and pain in the inner thigh (Ekpo et al., 2007; Redwine and Sharpe, 1990; Langebrekke and Qvigstad, 2009; Osorio et al., 2018; Waer et al., 2012; Fambrini et al., 2010; Kalkan and Daniilidis , 2018). These symptoms are reported as worsening during menstruation, making it easier to correlate it with extra pelvic endometriosis. Only one case reported nonspecific chronic pelvic pain (Peters et al., 2019).

Involvement of pelvic nerves by DIE implants is a less common occurrence, and among these, entrapment of obturator nerve due to DIE is extremely rare (<1%) (Kondo et al., 2013; Koninckx and Martin, 1994; Koninckx et al., 1991; Bradshaw and McCrory, 1997; Redwine and Sharpe, 1990; Langebrekke and Qvigstad, 2009; Osorio et al., 2018; Waer et al., 2012). Obturator involvement should always be considered in women of fertile age with cyclical pelvic and severe chronic inguinal pain that is localised to the inner thigh.

The patients’ medical history has great importance as their symptoms may be associated with extra pelvic localisations of the disease (12%) (Davis and Goldberg, 2017). However, in most cases, diagnosis is established using imaging techniques. As for other extra pelvic compartments, MRI seems to be the method of choice for assessing obturator nerve endometriosis (Lomoro et al., 2019).

According to this review, the diagnosis of all reported cases were made using MRI, with the exception of two in which the imaging techniques were not reported (Redwine and Sharpe, 1990; Peters et al., 2019).

In four of the eight previously reported cases, the obturator nerve involvement was caused by large endometriomas retracting or encasing the nerve (Ekpo et al., 2007; Osorio et al., 2018; Waer et al., 2012; Fambrini et al., 2010). These cases were easily diagnosed by imaging studies before surgery in combination with consistent signs and symptoms relating to specific nerve involvement. In the remaining four cases, endometriomas or specific symptoms of endometriosis were not observed before surgery. Obturator nerve involvement was either caused by retractile fibrotic tissue or macroscopically typical endometriotic tissue, which was visualised and dissected during surgery and later confirmed histologically (Redwine and Sharpe, 1990; Langebrekke and Qvigstad , 2009; Kalkan and Daniilidis , 2018; Peters et al., 2019).

An exceptional case was reported by Langebrekke and Qvigstad (2009). A 26- year-old patient with stage IB adenocarcinoma of the cervix underwent laparoscopic lymph node dissection of the iliac and obturator regions, internal cerclage, and vaginal trachelectomy. At the primary surgery, minimal peritoneal endometriosis was discovered over the bladder. At a later stage, the patient was diagnosed with obturator nerve endometriosis. It is possible that the primary surgery may have activated tissue factors and cytokines, which facilitated the endometriosis-only manifestation of the obturator nerve (Langebrekke and Qvigstad, 2009).

Management of extra pelvic endometriosis can be extremely complicated. Clinical manifestations may be controlled with gonadotrophin-releasing hormone agonists or continuous combined hormonal treatment. However, potential adverse effects or decreased efficacy with long-term administration make surgical excision, with histologic confirmation, the treatment of choice. Proper surgery of extra pelvic endometriosis requires a multidisciplinary team comprised of various specialists according to disease localisation. Treatment usually involves extraperitoneal exploration, surgical release of the nerve and wide excision of endometriotic tissue around the nerve. The functionality of laparoscopy in both diagnosis and treatment of endometriosis, together with the ability to access deep anatomic sites, makes it the preferred surgical option over laparotomy. The recurrence rate of endometriosis after surgery may be reduced by using ovarian function-inhibiting drugs (Jee et al., 2009).

In this review only one case received preoperative hormonal therapy (Waer et al., 2012). From the 8 reported obturator nerve endometriosis cases, 5 patients were treated laparoscopically (Ekpo et al., 2007; Langebrekke and Qvigstad, 2009; Fambrini et al., 2010; Kalkan and Daniilidis , 2018; Peters et al., 2019) and 3 cases underwent open surgery (Redwine and Sharpe, 1990; Osorio et al., 2018; Waer et al., 2012).

All cases benefited from laparoscopic or open surgical treatment with reports of almost complete recovery or improvement in symptoms in the early postoperative period. No damage to the nerve or any of its branches were reported. No sensory complaints, such as upper medial thigh pain or anaesthesia, were reported in any case. No additional hormonal therapy was used in any reported case during the postoperative period. The follow-up data was varying with none detailed for 3 of the cases (Redwine and Sharpe, 1990; Waer et al., 2012; Peters et al., 2019). For the other cases the duration of follow-up varied between 2 weeks (Ekpo et al., 2007), 3 months (Langebrekke and Qvigstad, 2009), 6 months (Osorio et al., 2018; Fambrini et al., 2010) and 18 months (Kalkan and Daniilidis, 2018). For all these cases, no recurrence of endometriosis or obturator nerve involvement symptoms were reported at their last follow-up visits (Ekpo et al., 2007; Redwine and Sharpe, 1990; Langebrekke and Qvigstad, 2009; Osorio et al.,2018; Waer et al., 2012; Fambrini et al., 2010; Kalkan and Daniilidis, 2018; Peters et al., 2019).

## Conclusion

Obturator nerve endometriosis is a rare condition. This condition should be treated with surgical exploration, as previously reported by others. The few reported cases demonstrate the invasive properties of endometriosis and its potential to involve the pelvic nerves. A thorough history of recurrent symptoms relating to the obturator nerve function, which worsens during menstrual cycles, alongside physical examination and appropriate radiologic imaging may lead to a diagnosis of obturator nerve involvement. The laparoscopic surgical approach is favourable for obturator nerve endometriosis and offers the advantage of precise discrimination of vital structures and good access to deep anatomic sites. Early diagnosis and treatment of obturator nerve endometriosis is important to minimise the cumulative nerve damage caused by recurrent cycles of bleeding and fibrosis, which are both characteristics of endometriosis.
